# Photocytotoxic Activity of Ruthenium(II) Complexes with Phenanthroline-Hydrazone Ligands

**DOI:** 10.3390/molecules26072084

**Published:** 2021-04-06

**Authors:** Priscila Pereira Silva-Caldeira, Antônio Carlos Almendagna de Oliveira Junior, Elene Cristina Pereira-Maia

**Affiliations:** 1 Departamento de Química, Centro Federal de Educação Tecnológica de Minas Gerais, Avenida Amazonas, 5253, Belo Horizontre (MG) 30421-169, Brazil; 2 Departamento de Química, Universidade Federal de Minas Gerais, Avenida Antônio Carlos, 6627, Belo Horizonte (MG) 31270-901, Brazil; acalmendagna@yahoo.com.br

**Keywords:** ruthenium, phenanthroline, hydrazone, antiproliferative activity, photocytotoxicity

## Abstract

This paper reports on the synthesis and characterization of two new polypyridyl-hydrazone Schiff bases, (*E*)-*N*′-(6-oxo-1,10-phenanthrolin-5(6*H*)-ylidene)thiophene-2-carbohydrazide (**L^1^**) and (*E*)-*N*′-(6-oxo-1,10-phenanthrolin-5(6*H*)-ylidene)furan-2-carbohydrazide (**L^2^**), and their two Ru(II) complexes of the general formula [RuCl(DMSO)(phen)(L^n^)](PF6). Considering that hydrazides are a structural part of severa l drugs and metal complexes containing phenanthroline derivatives are known to interact with DNA and to exhibit antitumor activity, more potent anticancer agents can be obtained by covalently linking the thiophene acid hydrazide or the furoic acid hydrazide to a 1,10-phenanthroline moiety. These ligands and the Ru(II) complexes were characterized by elemental analyses, electronic, vibrational, ^1^H NMR, and ESI-MS spectroscopies. Ru is bound to two different *N*-heterocyclic ligands. One chloride and one S-bonded DMSO in *cis*-configuration to each other complete the octahedral coordination sphere around the metal ion. The ligands are very effective in inhibiting cellular growth in a chronic myelogenous leukemia cell line, K562. Both complexes are able to interact with DNA and present moderate cytotoxic activity, but 5 min of UV-light exposure increases cytotoxicity by three times.

## 1. Introduction

Chemotherapy with cisplatin and related Pt-based drugs, despite being among the most effective anticancer drugs for a variety of tumors, presents several problems, such as acquired resistance [1], limited activity against some of the most common tumors, and severe side-effects [2,3]. Thus, complexes of transition metals other than Pt have been extensively investigated for their anticancer activity. Among the non-platinum compounds exhibiting anticancer properties, those of ruthenium have attracted interest for their favorable properties, such as the ability to bind nucleic acids and proteins, ligand exchange kinetics similar to platinum compounds, preferential accumulation in neoplastic tissues, ability to mimic iron binding in biological system, and lower toxicity than platinum compounds [4,5,6,7,8,9].

The ability of Ru(II) complexes with polypyridyl ligands to bind to DNA and proteins has been extensively investigated in an attempt to investigate their mechanism of cytotoxic action [10,11,12,13]. These complexes are also receiving great attention due to their potential in photodynamic therapy, which is an emerging anticancer treatment that comprises the presence of light, oxygen, and a photosensitizing drug to accomplish the photocytotoxic effect. The photoactivated drug produces reactive oxygen species, which can oxidize important cellular components such as DNA and cause cell death [13,14,15,16,17]. Ru(II) compounds of the type [Ru(L)_2_(sulfonamides)](PF_6_), in which L is either 2,2′-bipyridine or 1,10-phenanthroline, can bind and damage the BSA protein upon photoirradiation and the complex with 1,10-phenanthroline exhibited an increase in cytotoxicity against cancer cells of approximately 10 orders of magnitude upon photoactivation, showing potential to be used in photodynamic therapy (PDT) [18].

At the same time, another group of antitumor ruthenium compounds which have increasingly attracted the attention of researchers is ruthenium complexes with dimethylsulfoxide (DMSO) as ligand [19]. Ru(II) and Ru(III) complexes with coordinated dimethylsulfoxide have been shown to possess antitumor and antimetastatic properties [20,21,22,23]. NAMI-A (imidazolium *trans*-imidazoledimethylsulphoxidetetrachloridoruthenate), its sodium analogue or IT-139, and the indazolium trans-tetrachlorobis(1*H*-indazole)ruthenate(III) or KP1019, entered clinical trials for cancer [20,21,22,23,24]. NAMI-A was the first Ru-based drug entering a phase I study performed at the National Cancer Institute of Amsterdam (NKI) on patients suffering from different solid tumors [25]. Unfortunately, the occurrence of undesirable side effects caused the suspension of phase II trials using NAMI-A alone. Therefore, NAMI-A was entered into a phase I/II study in combination with gemcitabine as a second line treatment for metastatic non-small cell lung cancer. Because of the recurrence of side effects and the fact that it was less effective than gemcitabine alone, clinical trials were abandoned [26].

Another possibility to obtain new Ru complexes as potential anticancer agents concerns structural modifications in the ligand. Organometallic Ru(II) compounds containing different chelating ligands are promising anticancer agents [27]. Minor structural alterations in the structure of the compounds can affect their mechanism of action and cytotoxicities [28,29,30].

Hydrazones (–CO–NH–N=CH–) are an interesting class of compounds with a wide range of pharmacological applications [31], including anticancer activity [32,33,34].

We synthesized two new ligands possessing a phenanthrolyl group linked to a hydrazone moiety and their respective DMSO-Ru(II) complexes. Our strategy is to associate into a pro-ligand molecule a hydrazone moiety to a DNA intercalative agent. We have evaluated the complexes’ activities in a human tumor cell line, K562, without and with photoactivation.

## 2. Results and Discussion

### 2.1. Synthesis of the Phenanthroline-Hydrazone Ligands and Ru(II) Complexes

**Figure 1:**
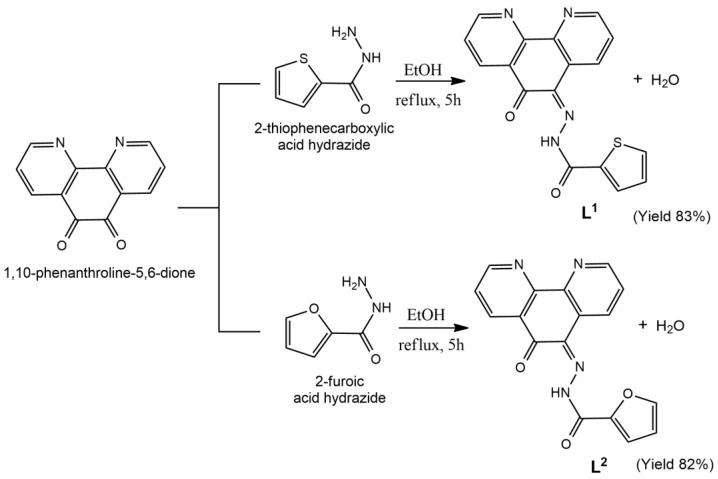
Reaction scheme showing the preparation of the ligands **L^1^** and **L^2^**.

Two new pyridyl-hydrazone Schiff-bases, **L^1^** and **L^2^**, were obtained in desirable purity and yield by condensation reactions of the 1,10-phenanthroline-5,6-dione with the appropriate acid hydrazide (Figure 1). The complexes **1** and **2** were obtained in moderate yields by treating each ligand with 1,10-phenanthroline (phen) and *cis*-[Ru(DMSO)_4_Cl_2_] in methanol, in the presence of NH_4_PF_6_.

**Figure 2:**
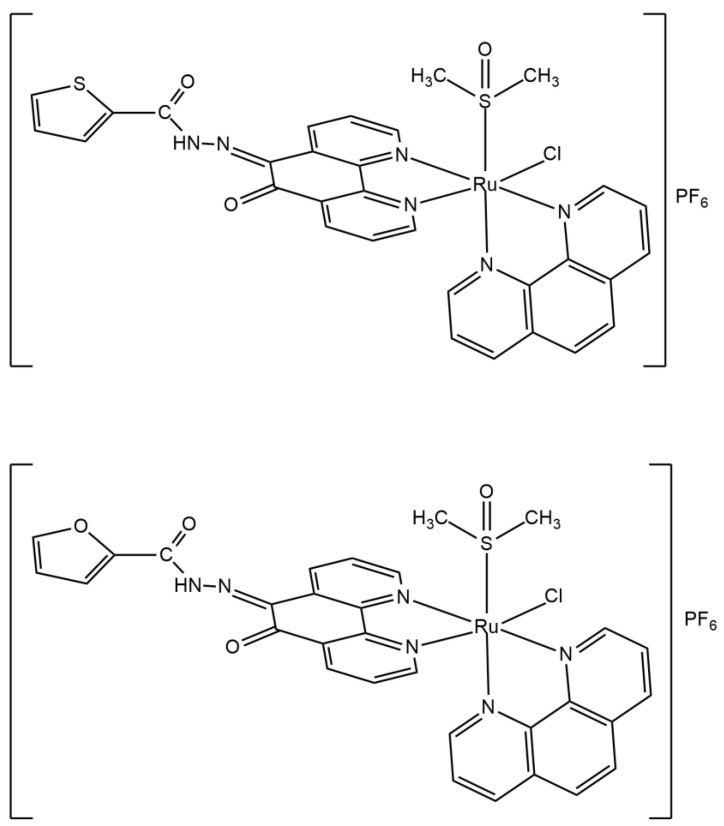
Chemical structures of complexes **1** (top) and **2** (bottom).

The results of elemental analysis are in accordance with the calculated values for **L^1^** (C_17_H_10_N_4_O_2_S), **L^2^** (C_17_H_10_N_4_O_3_), **1** ([RuCl(DMSO)(phen)(L^1^)]PF_6_), and **2** ([RuCl(DMSO)(phen)(L^2^)]PF_6_). Molar conductivity values for **1** and **2**, in nitromethane, are in the 1:1 electrolytes range [35], as expected for monocationic complexes. Ru is bound to the two phenanthroline ligands and to an S-bounded DMSO in *cis* position to the chloride. The structures of complexes **1** and **2** are shown in Figure 2. The *cis* geometry is favored by the trans-cooperative interaction of a π-donor ligand, such as Cl^−^, in trans position to a π -acceptor ligand, such as phen. Lacking a crystal structure, the complexes were characterized by elemental analyses, electronic, vibrational, ^1^H NMR, and ESI-MS spectroscopies.

**Figure 3:**
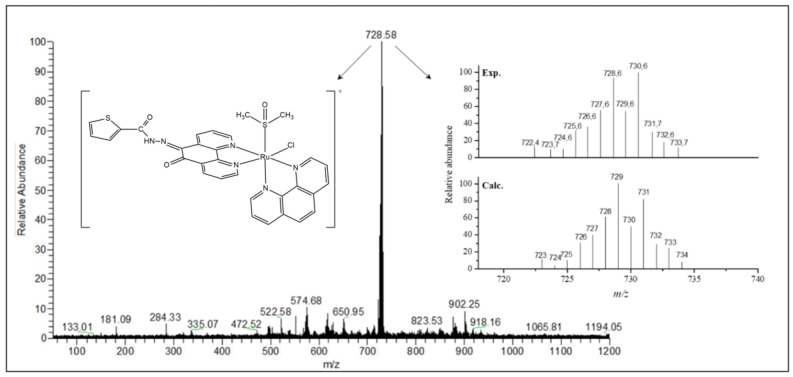
Electrospray ionization (ESI) spectrum of complex **1** in MeOH:H_2_O (50:50) solution and the isotopic distribution calculated for the species [Ru(Cl)(DMSO)(phen)(L^1^)]^+^ with the program Qual Browser 2.0.7copyright^®^ Thermo Fischer Scientific Inc.

**Figure 4:**
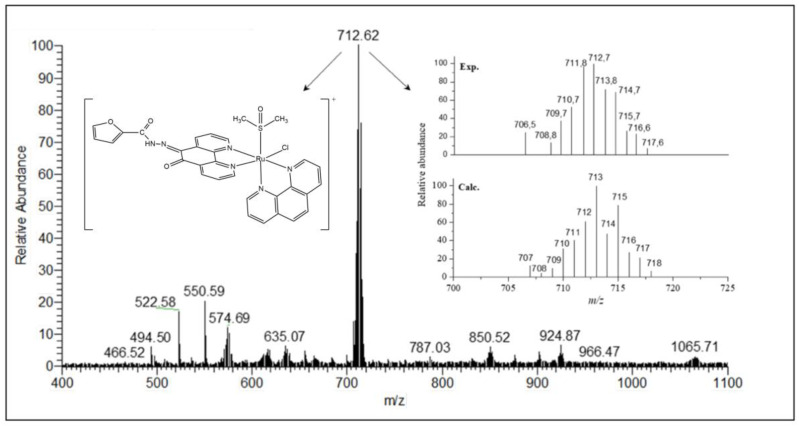
ESI spectrum of complex **2** in MeOH:H_2_O (50:50) solution and the isotopic distribution calculated for the species [Ru(Cl)(DMSO)(phen)(L^2^)]^+^ with the program Qual Browser 2.0.7copyright^®^ Thermo Fischer Scientific Inc.

Electrospray ionization mass spectrometry (ESI-MS) studies were carried out in the positive mode and in the range of *m*/*z* 150–1200. The mass spectra were measured in MeOH:H_2_O (50:50) solution and confirmed the formula proposed for all synthesized compounds. The ESI-MS spectrum of ligand **L^1^** presents two main peaks at *m*/*z* 690.63 (100%) and 335.04 (20%) (Figure S7). The *m*/*z* 690.63 corresponds to the dimeric species, [(C_17_H_10_N_4_O_2_S)_2_Na]^+^ (calculated mass 691.09) while the *m*/*z* 335.04 corresponds to the molecular ion peak, [(C_17_H_10_N_4_O_2_S)H]^+^ (calculated mass 335.06). For **L^2^** two main peaks are observed at *m*/*z* 319.04 (100%) and 658.68 (28%) (Figure S8). The first one corresponds to the molecular ion peak [(C_17_H_10_N_4_O_3_)H]^+^ (calculated mass 319.08), whereas the second indicates the presence of an Na^+^ adducted dimeric species [(C_17_H_10_N_4_O_3_)_2_Na]^+^ (calculated mass 659.14). The mass spectra of the Ru(II) complexes are in good agreement with the proposed molecular structures. The ESI spectra and calculated isotopic distribution patterns for complexes **1** and **2** are shown in Figure 3 and Figure 4. Two main peaks at *m*/*z* 728.58 (100%) and 712.62 (100%) for complexes **1** and **2**, respectively, exhibit the isotopic envelops of ruthenium and chlorine according to [RuCl(DMSO)(phen)(L^n^)]^+^, in which L^n^ = **L^1^** or **L^2^**, and calculated masses are 729.01 and 713.03 for **1** and **2**, respectively.

The assignments of the infrared spectra confirm the coordination of Schiff bases to Ru(II) through the heterocyclic nitrogen and the S-bonded coordination of the DMSO. A medium-sharp band around 1580 cm^−1^ due to the azomethine C=N stretching frequency is present in free ligands and complexes. Two intense bands appear in the region 1716–1626 cm^−1^ due to ν(C=O) of the carbonyl groups present in **L^1^** and **L^2^** (Figures S1 and S2). The presence of νC=N and νC=O indicates that the ligands exist in the amide form in the solid state. For both complexes, infrared spectra show that the DMSO ligands are S-bonded to ruthenium(II) (Figures S9 and S10), since the band observed at 424 cm^−1^ is attributed to ν(Ru–S) [36]. The characteristic P–F stretch of the PF_6_ counterion was seen at 842 and 840 cm^−1^ for complexes **1** and **2**, respectively, whereas the δ(PF_6_) bending vibrations in the spectra of both complexes are observed as a narrow and strong band at 558 cm^−1^ [36].

**Figure 5:**
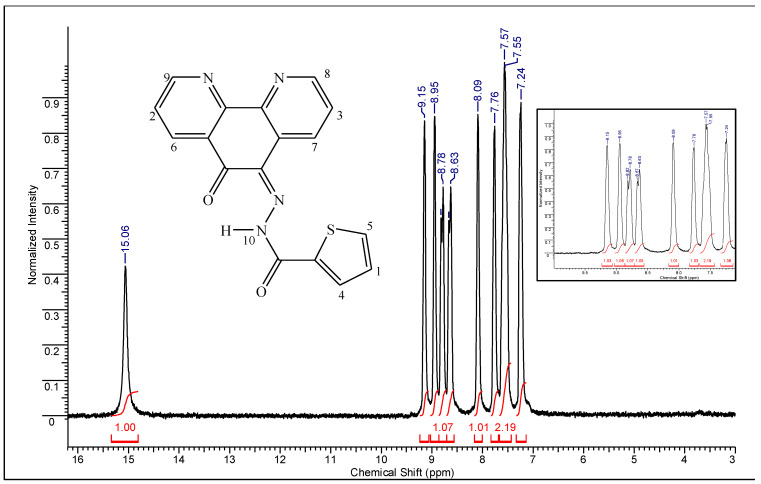
^1^H NMR spectrum of **L^1^** in CDCl_3_.

The ^1^H NMR spectra of the ligands and the complexes were recorded to confirm the formation of the Schiff base ligands and their Ru(II) complexes. Both ligands exhibited quite similar ^1^H NMR spectra in CDCl_3_; the spectrum of the **L^1^** is shown in Figure 5. A singlet at δ 15.0 and 15.4, in the spectra of **L^1^** and **L^2^**, respectively, has been assigned to the N–H proton. The multiple signals observed in the range δ 6.5–9.2 correspond to the presence of nine aromatic hydrogens for each ligand, which is in accordance with the proposed structures (Figures S3–S6 and Table S1). The ^1^H NMR spectra of the complexes recorded in DMSO-*d*_6_ comprise neat signals characteristic of diamagnetic complexes, which confirm the ruthenium oxidation state as 2^+^. The ^1^H NMR spectra of complexes **1** and **2** are slightly different (Figures S11 and S12). In the region 6.6–9.3 the signals of aromatic protons from phenanthroline and **L^1^** or **L^2^** appear overlapped. The presence of 17 aromatic hydrogens is in accordance with the stoichiometry of 1phen:1Ru:1L^n^. The signal observed around δ 2.5 in both complexes’ spectra is due to the residual solvent. No peaks are observed at δ 2.7, ruling out the possibility of O-coordination of DMSO in the complexes [37]. The group of peaks around δ 2.9–3.4 ppm is due to methyl resonances of the S-bonded DMSO ligand, which is supported by IR spectra analysis. The presence of water in DMSO-*d*_6_ also contributes to the intense signal at δ 3.3. The coordination of the hydrazone moiety would involve the removal of the N–H proton and enolization originating an imidolate oxygen [38]. Thus, the non-participation of the acid hydrazone group in Ru(II) coordination can be confirmed by the presence of the singlet at 14.8 and 15.2 ppm, for **1** and **2** respectively, attributed to N–H. The appearance of very small peaks in the region 9.8–10.4 ppm indicates the presence of free ligands resulting from solvolysis of the complexes in DMSO.

**Figure 6:**
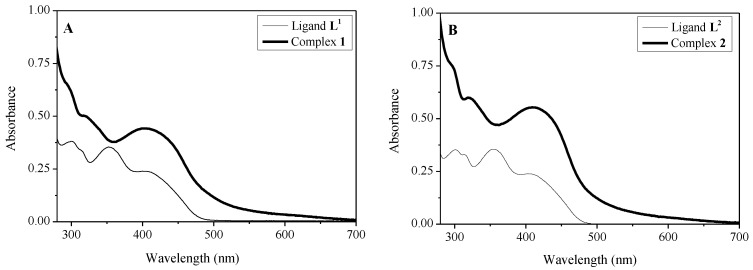
Electronic spectra of DMSO solutions of the synthetized ligands and complexes at 2.5 × 10^−4^ mol L^−1^, l = 1 cm. (**A**) ligand **L^1^** and complex **1** and (**B**) ligand **L^2^** and complex **2**.

The electronic spectra of the free Schiff bases and corresponding Ru(II) complexes were recorded in DMSO (Figure 6). The spectra of the phenanthrolyl hydrazones display three bands due to π-π* transitions of the aromatic system and n-π* transitions of the carbonyl (C=O) and azomethine (C=N) groups [38]. A broad and intense absorption band at 410 nm appears in the spectra of the complexes due to a dπ-π* metal to ligand charge transfer (MLCT) transition overlapped with the less intense n-π* transitions of the carbonyl (C=O) and azomethine (C=N) groups. The pattern of the electronic spectra of the complexes indicated an octahedral environment around the Ru(II) ion [39].

### 2.2. Evaluation of Cytotoxic and Photocytotoxic Effect

The effect of the ligands and ruthenium complexes on the growth of tumoral cells was studied in a chronic myelogenous leukemia cell line (K562). The newly synthesized hydrazones exhibit high activity against chronic myelogenous leukemia cells. The IC_50_ values, at concentrations required to inhibit 50 % of cell growth, are shown in Table 1. The dose-response curves are given in Figure S13. Under the same experimental conditions, *cis*[RuCl_2_(phen)_2_] has no effect on cellular growth up to 100 μmol L^−1^. For the sake of comparison, acylhydrazone derivatives containing furan synthesized as anticancer agents inhibit the growth of a human promyelocytic leukemia cell line (HL-60) with IC_50_ values ranging from 16.4 to 125 μmol L^−1^ [40].

The ruthenium complexes **1** and **2** inhibit the growth of leukemia cells in a concentration-dependent manner, but with a cytotoxic activity inferior to those of the free ligands. However, the possibility to exploit the intense metal-to-ligand charge transfer transitions makes the Ru(II) complexes potential PDT agents.

With the aim of investigating the influence of irradiation on the cytotoxicity of the complexes, we have first examined if UV-A exposure had an effect in the complexes by following their mass spectra before and after irradiation. After 5, 15, and 30 min of UV-A irradiation, the MS spectra of **1** and **2** remain unchanged, with the same characteristic peaks observed under dark conditions (Figures S14 and S15). Therefore, the cytotoxic effect was determined by irradiating the cells with UV-A light for 5 min after being incubated with compounds for 4 h. The IC_50_ values obtained in the dark and after 5 min of UV-A irradiation are shown in Table 1 and the dose-response curves in Figure S16. UV-light exposure enhanced the activity of both complexes, with a photocytotoxic index, IC_50 dark_/IC_50 irradiated_, around 3.

### 2.3. DNA Interaction

The interactions between the complexes and calf thymus DNA were studied by UV–Vis spectrophotometry. A representative experiment obtained with complex **2** at 6.0 × 10^−5^ M and [DNA] varying from 0 to 2 × 10^−4^ M is shown in Figure 6. One can observe that the addition of DNA induces a hypochromic effect and a minor bathochromic shift in the spectra of the ruthenium complex, indicating its interaction with DNA. The binding constant, K, was calculated from the spectrophotometric data accordingly to the equation

[DNA]/(ε_a_ − ε_f_) = [DNA]/(ε_0_ − ε_f_) + 1/K(ε_0_ − ε_f_) (1)

in which [DNA] is the concentration of DNA in base pairs, ε_a_ is the ratio of Abs/[Ru], ε_f_ is the extinction coefficient of the free Ru(II) complex and ε_0_ is the extinction coefficient of the complex in the fully bound form, at 410 nm. A plot of [DNA]/(ε_a_ − ε_f_) versus [DNA] gives a straight line whose slope is 1/(ε_0_ − ε_f_) and the *Y* intercept is equal to 1/K(ε_0_ − ε_f_). The binding constants, obtained from the ratio of the slope to the *Y* intercept, are 5.5 × 10^4^ and 9.0 × 10^3^ for complexes **1** and **2**, respectively. The substitution of an oxazole for the thiazole ring diminished DNA binding affinity.

For the sake of comparison, binding constants for Ru(II) complexes containing N-heterocyclic ligands vary from 7 × 10^2^ for [Ru(bpy)_3_]^2+^ and [Ru(bpy)_2_phen]^2+^; 2.3 × 10^3^ for [RuCl_2_(DMSO)_2_(tcah)], where tcah = thiophene-2-carboxylicacid hydrazide; 3.4 × 10^4^ for [RuCl(phen)_2_(DMSO)]^2+^; 0.15–3.2 × 10^6^ for [Ru(phen)_2_(dppz)]^2+^ and [Ru(bpy)_2_(dppz)]^2+^; to ~10^8^ for [Ru(phen)_2_dppz]^2+^. The nature of the polypyridyl ligand and the presence of a leaving group in the metal ion affect DNA binding affinity. Complexes **1** and **2** exhibit a moderate DNA affinity inferior to substitutionally inert polypyridyl complexes, which are uniquely DNA intercalators [5,37,41,42].

## 3. Materials and Methods

RuCl_3_·xH_2_O, 1,10-phenanthroline monohydrate (phen·H_2_O), 2-thiophenecarboxylic acid hydrazide, 2-furoic acid hydrazide, and NH_4_PF_6_ were purchased from Sigma-Aldrich (Sigma, Saint Louis, MO, USA). All other chemicals were reagent grade and were used without further purification. Calf thymus DNA sodium salt (CT DNA) was used as obtained from Sigma Co. (Sigma, Saint Louis, MO, USA).

Carbon, nitrogen, and hydrogen were determined on a Perkin–Elmer 2400 CHN analyzer (Perkin Elmer Inc., Waltham, MA, USA). Conductivity measurements were carried out with a Digimed DM 31 conductivity meter (Digimed, São Paulo, Brazil) using a cell of constant 1.013 cm^−1^. The solvent used was spectroscopic grade nitromethane (Merck, Darmstadt, Germany) (ΛM = 8.80 S cm^2^ mol^−1^) and Me_4_NBr (ΛM = 102.20 S cm^2^ mol^−1^) was used as standard. Infrared spectra were recorded over the region 400–4000 cm^−1^ in a Perkin–Elmer 283 B spectrometer (Perkin Elmer Inc., Waltham, MA, USA). The samples were examined in KBr pellets. Full scan mass spectra (MS mode) were obtained on a MicroTOF LC Bruker Daltonics spectrometer (Bruker Daltonics Fremont, CA, USA) equipped with an electrospray source operating in positive ion mode. Samples were dissolved in an MeOH:H_2_O (50:50) solution and were injected into the apparatus by direct infusion. Tandem mass spectra (MS^2^) were achieved by collision-induced dissociation (CID) and were obtained by selecting a given complex ion into the collision cell using argon as collision gas. ^1^H NMR spectra were obtained in Bruker Advance DRX 200 spectrometer (Billerica, MA, USA) using CDCl_3_ or DMSO-*d*_6_ with TMS as an internal standard. A Cary100 Varian spectrometer (Santa Clara, CA, USA) was used for UV–Vis absorption spectroscopy. For the interactions with calf thymus DNA (CT DNA), the complex concentration used was 6 × 10^−5^ and the DNA: complex molar ratios varied from 0 to 4. The DNA concentration per nucleotide was determined by the ε = 6600 M^−1^ cm^−1^ at 260 nm. The ionic strength was maintained constant with 1 × 10^−3^ M NaCl and the pH was fixed at 7.2 with 20 mM Tris-HCl buffer. The absorbance of DNA itself was subtracted by adding an equal quantity of DNA to both the complex and the reference solutions.

*Synthesis of the ligands:* The precursor 1,10-phenthroline-5,6-dione (pdo) was prepared from a modification of the literature method [43]. A reflux system and a thermometer were attached to a three-neck round-bottom flask, to which 5.0 g of 1,10-phenanthroline and 30 mL of concentrated sulfuric acid were added. For complete solubilization of phen·H_2_O, the system was heated (60 °C) for 10 min. Heating was stopped and 2.5 g of NaBr was added to the resulting solution. Following the solubilization of NaBr, 17 mL of concentrated HNO_3_ were added to the solution, which remained at reflux for 50 min. After that time, the solution was left to reach room temperature. Then the solution was transferred into a beaker and placed in an ice bath for neutralization with an NaOH solution (10 mol L^−1^) until pH 6.9. The suspension was then filtered under a vacuum. Under constant stirring, 100 mL of CH_2_Cl_2_ was added to the solution. The resulting mixture was transferred to a separatory funnel and the organic extract was separated. To the organic extract, 2.0 g of Na_2_SO_4_ previously dried in an oven were added to eliminate any residual water present in this solution. The solvent of the organic extract was removed with the aid of a rotatory evaporator and subsequently 100 mL of MeOH were added to the residue. The MeOH solution was then transferred to a beaker and placed in the refrigerator (~4n °C). On the following day, yellow-orange needle crystals were separated from the solution by simple filtration and washed with ether. Elemental analysis of these crystals confirmed that this was pdo.

The Schiff bases were prepared using a general procedure by the reaction of pdo and the hydrazide (2-thiophenecarboxylic acid hydrazide-L^1^ or 2-furic hydrazide acid-L^2^), as shown in Figure 1. Both ligands were prepared in the same way, therefore only the preparation of **L^1^** is described below.

In a round-bottom flask coupled with a reflux system, 1,10-phenanthroline-5,6-dione (1.0 mmol, 0.210 g) and 2-thiophenecarboxylic acid hydrazide (1.2 mmol, 0.171 g) for **L^1^** or 2-furoic acid hydrazide (1.2 mmol, 0.151 g) for **L^2^** were dissolved in EtOH (30 mL) and 1.0 mL of glacial acetic acid. The reaction mixture was refluxed for 5 h. In a rotary evaporator, the volume of the solution was reduced by half. The formed precipitate was separated by vacuum filtration, washed thoroughly with cold ethyl ether and dried, giving an 83% yield.

For the preparation of **L^2^**, the compound 2-furoic acid hydrazide was added instead of 2-thiophenecarboxylic acid hydrazide, giving an 82% yield.

*(E)-N′-(6-oxo-1,10-phenanthrolin-5(6H)-ylidene)thiophene-2-carbohydrazide (Ligand **L^1^**)*: 0.278 g (0.83 mmol, 83%); M.P. 225 (± 3) °C. Anal. Calc. for C_17_H_10_N_4_O_2_S (334.35 g mol^−1^): C, 61.07; H, 3.01; N, 16.76%. Found: C, 61.29; H, 2.71; N, 16.59%. IR (KBr, ν_max_, cm^−1^): 3030, 1666, 1626, 1580, 1558, 1496, 1462, 1412, 1354, 1332, 1290, 1224, 1196, 1126, 1084, 1064, 1014, 812, 800, 770, 730, 718, 692, 620, 590, 444. ESI-MS (+) (CH_3_OH:H_2_O): *m*/*z*= 690.63 (100%, [(C_17_H_10_N_4_O_2_S)_2_Na]^+^), *m*/*z*_Calc._ = 691.09; *m*/*z* = 335.04 (20%, [(C_17_H_10_N_4_O_2_S)H]^+^), *m*/*z*_Calc._ = 335.06. UV-vis (DMSO) λ_max_ nm (ε, M^−1^ cm^−1^): 301 (1480), 355 (1440), and 410 (920). ^1^H NMR (200 MHz, CDCl_3_, δ ppm): 7.24 (d, C_20_–H), 7.56 (d, C_2_–H, C_13_–H), 7.76 (s, C_21_–H), 8.09 (s, C_24_–H), 8.65 (d, C_1_–H), 8.80 (d, C_14_–H), 8.95 (s, C_12_–H), 9.15 (s, C_3_–H), 15.06 (s, N_17_–H). Color: yellow.

*(E)-N′-(6-oxo-1,10-phenanthrolin-5(6H)-ylidene)furan-2-carbohydrazide (Ligand **L^2^**)*: 0.261 g (0.82 mmol, 82%); M.P. 240 (± 3) °C. Anal. Calc. for C_17_H_10_N_4_O_3_ (318.29 g mol^−1^): C, 64.15; H, 3.17; N, 17.60%. Found: C, 61.1; H, 3.1; N, 16.9%. IR (KBr, ν_max_, cm^−1^): 3408, 1716, 1628, 1578, 1506, 1468, 1414, 1382, 1292, 1264, 1234, 1184, 1148, 1064, 1016, 918, 882, 816, 778, 748, 732, 708, 606. ESI-MS (+) (CH_3_OH:H_2_O): *m*/*z* = 319.04 (100%, [(C_17_H_10_N_4_O_3_)H]^+^), *m*/*z*_Calc._ = 319.08; *m*/*z* = 658.68 (28%, [(C_17_H_10_N_4_O_3_)_2_Na]^+^), *m*/*z*_Calc._ = 659.14. UV-vis (DMSO) λ_max_ nm (ε, M^−1^ cm^−1^): 301 (1480), 355 (1440), and 410 (920). ^1^H NMR (200 MHz, CDCl_3_, δ ppm): 6.69 (d, C_20_–H), 7.27 (d, C_2_–H, C_13_–H), 7.52 (s, C_21_–H), 7.74 (s, C_24_–H), 8.69 (d, C_1_–H), 8.86 (d, C_14_–H), 8.97 (s, C_12_–H), 9.18 (s, C_3_–H), 15.47 (s, N_17_–H). Color: yellow.

*Synthesis of the complexes:* The complexes were synthesized using the same general procedure. The precursor *cis*-[Ru(DMSO)_4_Cl_2_] was prepared according to a literature method [19]. *cis*-[Ru(DMSO)_4_Cl_2_] (0.25 mmol, 0.121 g) and **L^1^** (0.25 mmol, 0.084 g) or **L^2^** (0.25 mmol, 0.080 g) were heated under reflux in MeOH (30 mL) for 60 min. Then a methanolic solution (6 mL) containing phen·H_2_O (0.25 mmol, 0.045 g) and NH_4_PF_6_ (0.50 mmol, 0.081g) was added and the resultant mixture was refluxed for three more hours. The precipitate was filtered, washed with EtOH, and dried under vacuum.

*[RuCl(DMSO)(phen)(L^1^)]PF_6_ (complex **1**)*: Yield: 0.109 g of dark red material (0.120 mmol, 48%). Anal. Calc. for [Ru(C_17_H_10_N_4_O_2_S)(C_12_H_8_N_2_)((CH_3_)_2_SO)Cl]PF_6_·2H_2_O (910.15 g mol^−1^): C, 40.91; H, 3.10; N, 9.61 %. Found: C, 41.09; H, 2.58; N, 9.63%. IR (KBr, ν_max_, cm^−1^): 3444, 3084, 1692, 1668, 1640, 1588, 1504, 1428, 1410, 1354, 1290, 1230, 1230, 1200, 1072, 1032, 968, 842, 770, 722, 684, 596, 558, 524, 426. ΛM = 89.6 S cm^2^ mol^−1^ in nitromethane. ESI-MS(+) (CH_3_OH:H_2_O): *m*/*z* = 728.58 (100%, [RuCl(DMSO)(phen)(L^1^)]^+^), C_31_H_24_ClN_6_O_3_RuS_2_, *m*/*z*_Calc._ = 729.01. UV-vis (DMSO) λ_max_ nm (ε, M^−1^ cm^−1^): 410 (1760).

*[RuCl(DMSO)(phen)(L^2^)]PF_6_ (complex **2**)*: Yield: 0.116 g of dark red material (0.130 mmol, 52%). Anal. Calc. for [Ru(C_17_H_10_N_4_O_3_)(C_12_H_8_N_2_)((CH_3_)_2_SO)Cl]PF_6_·2H_2_O (894.09 g mol^−1^): C, 41.64; H, 3.16; N, 9.40%. Found: C, 41.91; H, 2.81; N, 9.74%. IR (KBr, ν_max_, cm^−1^): 3362, 3084, 2970, 1694, 1644, 1580, 1506, 1486, 1428, 1382, 1290, 1268, 1236, 1188, 1110, 1072, 1028, 1018, 924, 840, 772, 720, 684, 558, 524, 424. ΛM = 77.9 S cm^2^ mol^−1^ in nitromethane. ESI-MS(+) (CH_3_OH:H_2_O): *m*/*z* = 712.62 (100%, [RuCl(DMSO)(phen)(L^2^)]^+^), C_31_H_24_ClN_6_O_4_RuS, *m*/*z*_Calc._ = 713.03. UV-vis (DMSO) λ_max_ (ε, M^−1^ cm^−1^): 410 (2160).

*Cells and culture:* The K562 cell line was purchased from the Rio de Janeiro Cell Bank (number CR083 of the RJCB collection). This cell line was established from pleural effusion of a 53-year-old female with chronic myelogenous leukemia in terminal blast crisis. Cells were cultured in RPMI 1640 (Sigma Chemical Co.) medium supplemented with 10% fetal calf serum (CULTILAB, São Paulo, Brazil) at 37 °C in a humidified 5% CO_2_ atmosphere. Cultures grew exponentially from 10^5^ cells mL^-1^ to about 8 × 10^5^ cells mL^-1^ in three days. Cell viability was checked by Trypan Blue exclusion. The cell number was determined by Coulter counter analysis.

For cytotoxicity assessment, 1 × 10^5^ cells mL^−1^ were cultured for 72 h in the absence and presence of various concentrations of tested compounds. For photocytotoxicity assessment, 1 × 10^5^ cells mL^−1^ were cultured in the dark for 4 h in the absence and the presence of a range of concentrations of tested compounds. Subsequently, cells were washed three times with ice-cold phosphate-buffered saline (PBS) to eliminate the culture medium. After replacement of the culture medium with PBS, the cells were photoirradiated with UV-A light (365 nm, 610 µW cm^−2^) for 5 min in a Spectroline Model CX-20—ultraviolet fluorescence analysis cabinet. After irradiation, PBS was replaced with RPMI 1640 medium supplemented with 10% fetal calf serum, and incubation was continued for a further 72 h in the dark. For cytotoxic and photocytotoxic assays, the sensitivity to compounds was evaluated by the concentration that inhibits cell growth by 50%, IC_50_. Stock solutions of the compounds were prepared in DMSO.

## 4. Conclusions

As hydrazides are a structural part of several drugs and metal complexes containing phenanthroline derivatives interact with DNA and exhibit antitumor activity, new compounds were prepared by linking the thiophene acid hydrazide or the furoic acid hydrazide to a 1,10-phenanthroline moiety. Two Ru(II) complexes with the new ligands were synthesized and characterized. This strategy resulted in ligands and Ru(II) complexes with high cytotoxicity against myelogenous leukemia cells. The metal complexes interact with DNA, inhibit the growth of leukemia cells in a concentration dependent manner, and the cytotoxicity increases three times after UV-A irradiation. The possibility to exploit metal-to-ligand charge transfer transitions makes the Ru(II) complexes of interest as PDT agents.

## Figures and Tables

**Table 1:** Growth inhibition of K562 cells by compounds **1** and **2** in dark and irradiated conditions.

Compound	IC_50_^a^ (μmol L^−1^ ± s.d.)			Photocytotoxic Index ^b^
	72 h (Dark)	4 h (Dark)	4 h (Irradiated)	
**L^1^**	2.59 ± 0.26	-	-	-
**L^2^**	2.06 ± 0.21	-	-	-
**1**	36.13 ± 3.6	55.06 ± 5.5	18.82 ± 1.9	2.9
**2**	35.80 ± 3.6	51.81 ± 5.2	17.26 ± 1.8	3.0
*cis*-[RuCl_2_(phen)_2_] ^c^	>100	-	-	-

^a^ IC_50_ is the concentration required to inhibit 50% of cell growth, after 3 days or 4 h of incubation in dark conditions or 4 h of incubation followed by 5 min of UV-A light exposure. The values are the mean of triplicate determinations. ^b^ Photocytotoxic index is the ratio of IC_50_ in the dark to IC_50_ after light irradiation. ^c^ Data from reference [18].

## Data Availability

Data is contained within the article or supplementary material.

## Supplementary Materials

The following are available online: Figure S1. Infrared spectrum of **L^1^** in KBr disks; Figure S2. Infrared spectrum of **L^2^** in KBr disks; Figure S3. ^1^H NMR spectrum of **L^1^** (200 MHz) in CDCl_3_; Figure S4. Section of the ^1^H NMR spectrum of **L^1^** (200 MHz) in CDCl_3_; Figure S5. ^1^H NMR spectrum of **L^2^** (200 MHz) in CDCl_3_; Figure S6. Section of the ^1^H NMR spectrum of **L^2^** (200 MHz) in CDCl_3_; Table S1. ^1^H RMN data of the ligands **L^1^** and **L^2^**; Figure S7. ESI-MS spectrum of **L^1^** in MeOH:H_2_O (1:1); Figure S8. ESI-MS spectrum of **L^2^** in MeOH:H_2_O (1:1); Figure S9. Infrared spectrum of complex **1**; Figure S10. Infrared spectrum of complex **2**; Figure S11. ^1^H NMR spectrum of complex **1** (200 MHz) in DMSO-*d*_6_; Figure S12. 1H NMR spectrum of complex **2** in DMSO-*d*_6_; Figure S13. Mass spectra (ESI-MS) of **1** before (A) and after UV-A light irradiation (B. C. D) for 5, 15, and 30 min. respectively; Figure S14. Mass spectra (ESI-MS) of **2** before (A) and after UV-A light irradiation (B. C. D) for 5, 15, and 30 min. respectively. Figure S15. Mass spectra (ESI-MS) of complex **2** before (A) and after UV-A light irradiation (B, C, D) for 5, 15, and 30 min, respectively. Figure S16. Photocytotoxic assays for complex **1** and complex **2**. K562 cells were incubated with different concentrations of tested compound for 4 h in the presence of different complex concentrations, in the dark and after 5 min of UV-A light exposure. The values are the average of three independent experiments.

## Sample Availability

Samples of the compounds are available from the authors.
