# Supplementary material for: Photocytotoxic Activity of Ruthenium(II) Complexes with Phenanthroline-Hydrazone Ligands
Source: Molecules. 2021 Apr 6;26(7):2084. doi: 10.3390/molecules26072084 (PMC8038675; doi:10.3390/molecules26072084)
Supplement: Supplementary file 1 [file molecules-26-02084-s001.pdf]

molecules

MDPI

Supplementary Materials

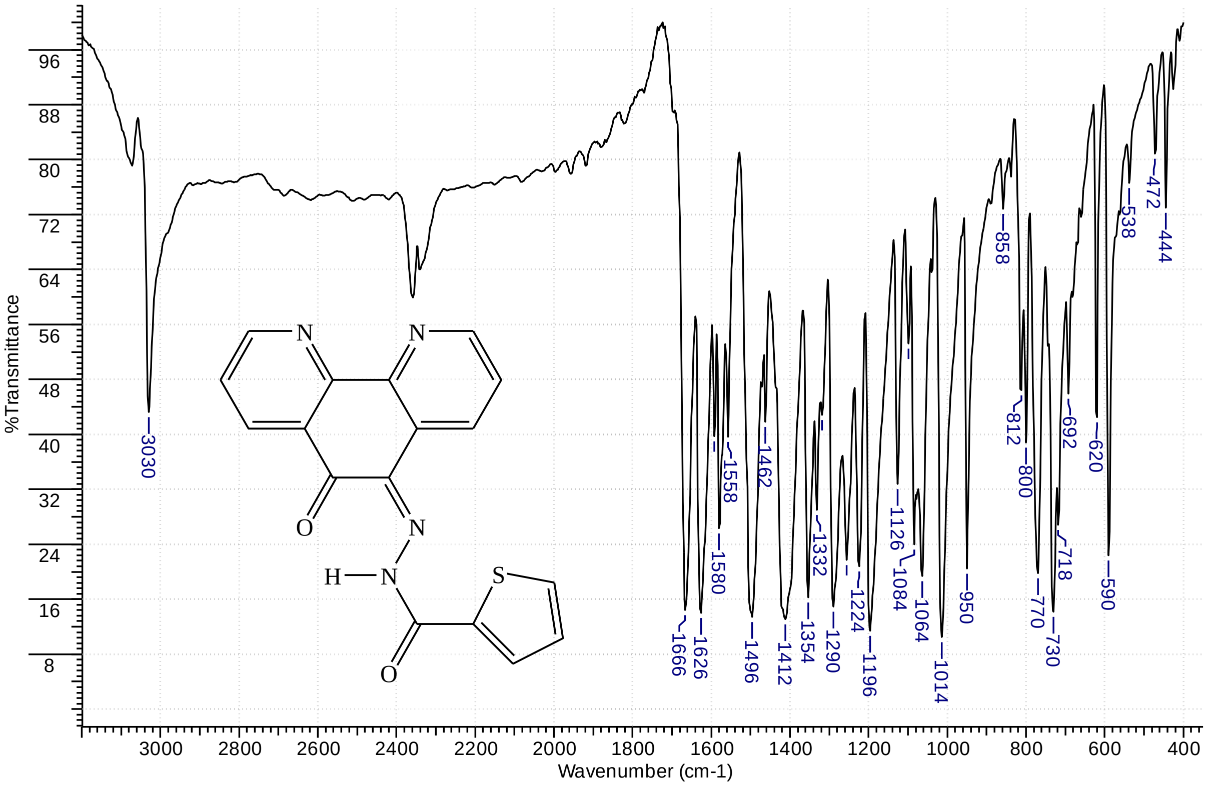

Figure S1. Infrared spectrum of $\mathbf{L}^1$ in KBr disks.

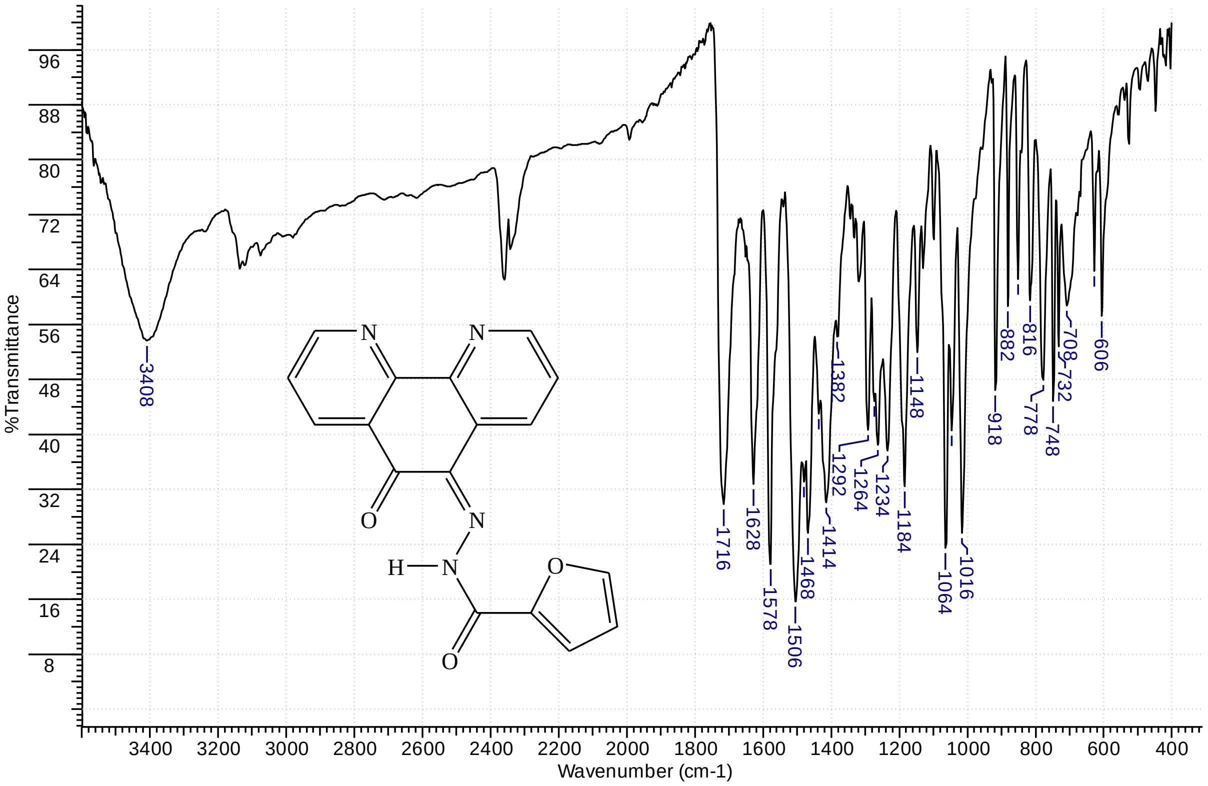

Figure S2. Infrared spectrum of **L**^{2} in KBr disks.

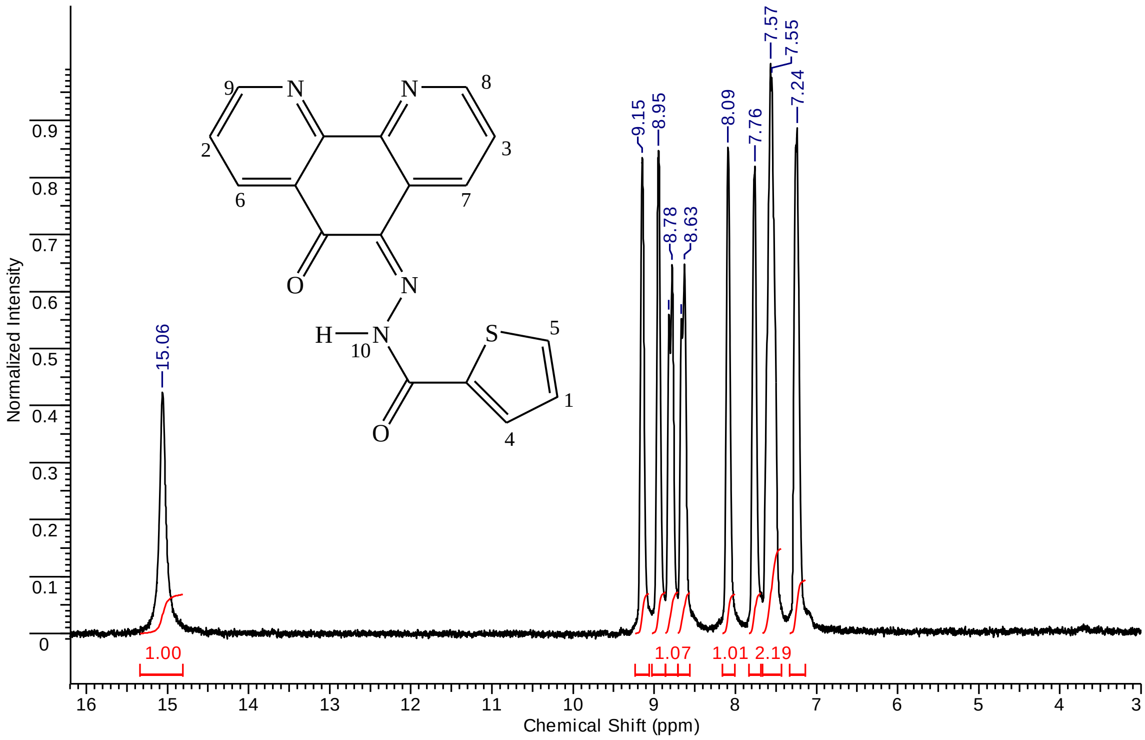

Figure S3. ^{1}H NMR spectrum of **L¹** (200 MHz) in $CDCl_3$.

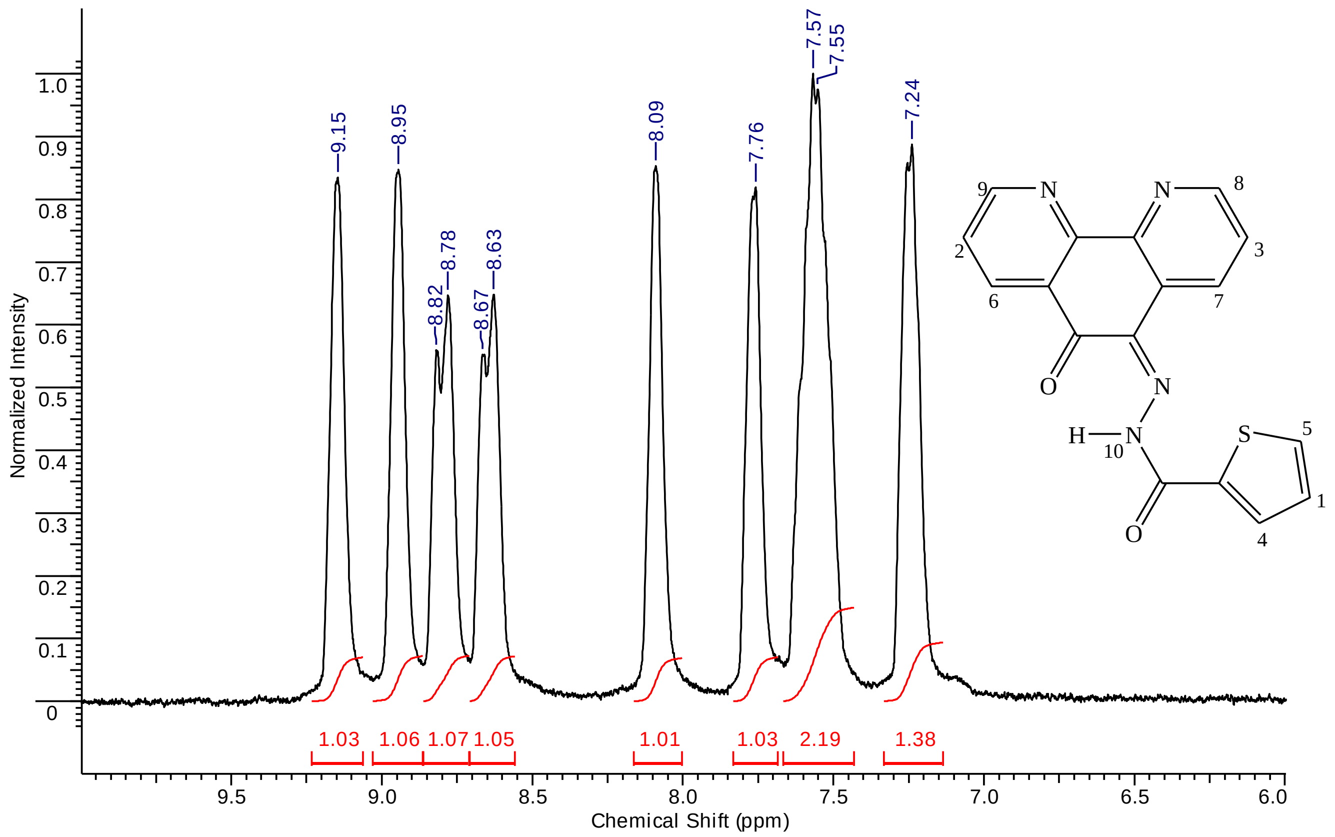

Figure S4. Section of the ^{1}H NMR spectrum of **L**^{1} (200 MHz) in $CDCl_3$.

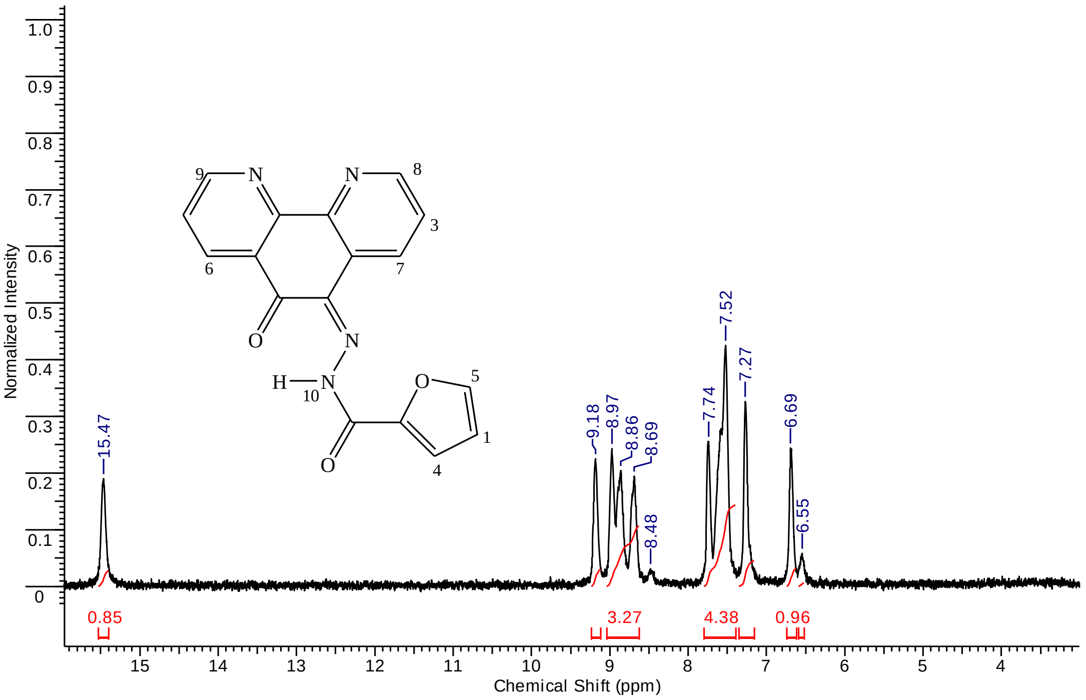

Figure S5. ^{1}H NMR spectrum of $\mathbf{L^2}$ (200 MHz) in $CDCl_3$.

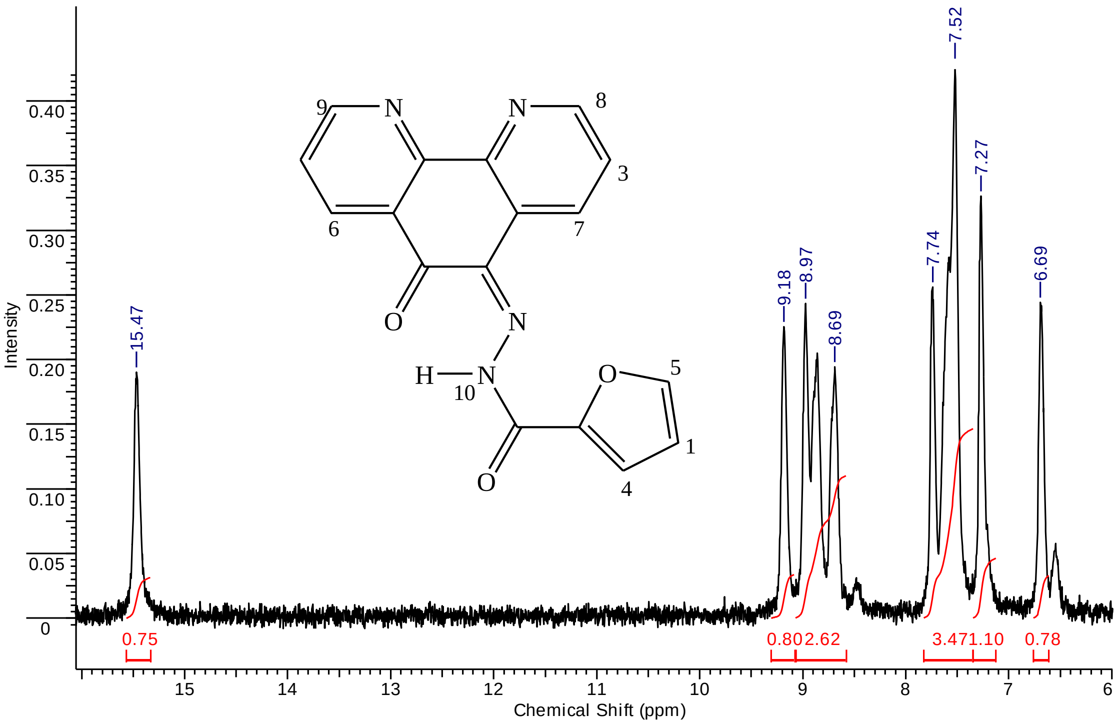

Figure S6. Section of the ^{1}H NMR spectrum of $\mathbf{L^2}$ (200 MHz) in $CDCl_3$.

Table S1. ^{1}H NMR data of the ligands **L^1** and **L^2**.

L^1			L^2		
δ (ppm)	Integral	Assignments	δ (ppm)	Integral	Assignments
7.24	1.38	H1	6.69	0.78	H1
7.56	2.19	H2, H3	7.27	1.1	H4
7.76	1.03	H4	7.6	3.47	H3, H2, H5
8.09	1.01	H5			
8.65	1.05	H6			
8.80	1.07	H7	8.92	2.62	H6, H7, H8
8.95	1.06	H8			
9.15	1.03	H9	9.18	0.80	H9
15.06	1.00	H10	15.47	0.75	H10

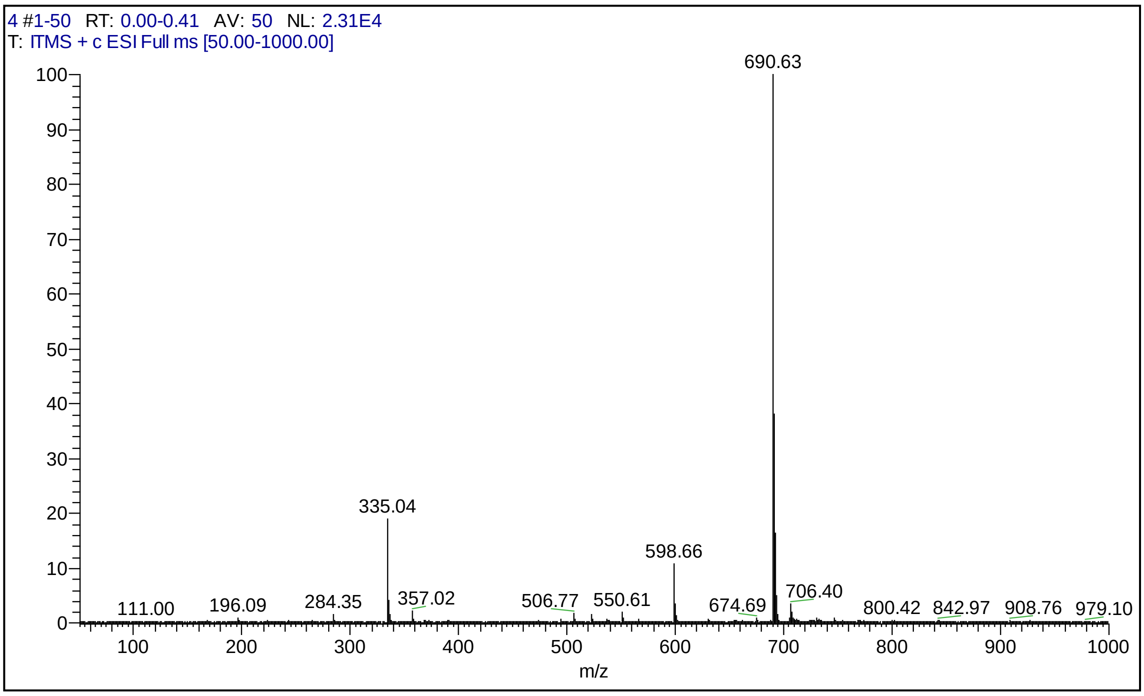

Figure S7. ESI-MS spectrum of **L**[1] in MeOH:H_2O (1:1).

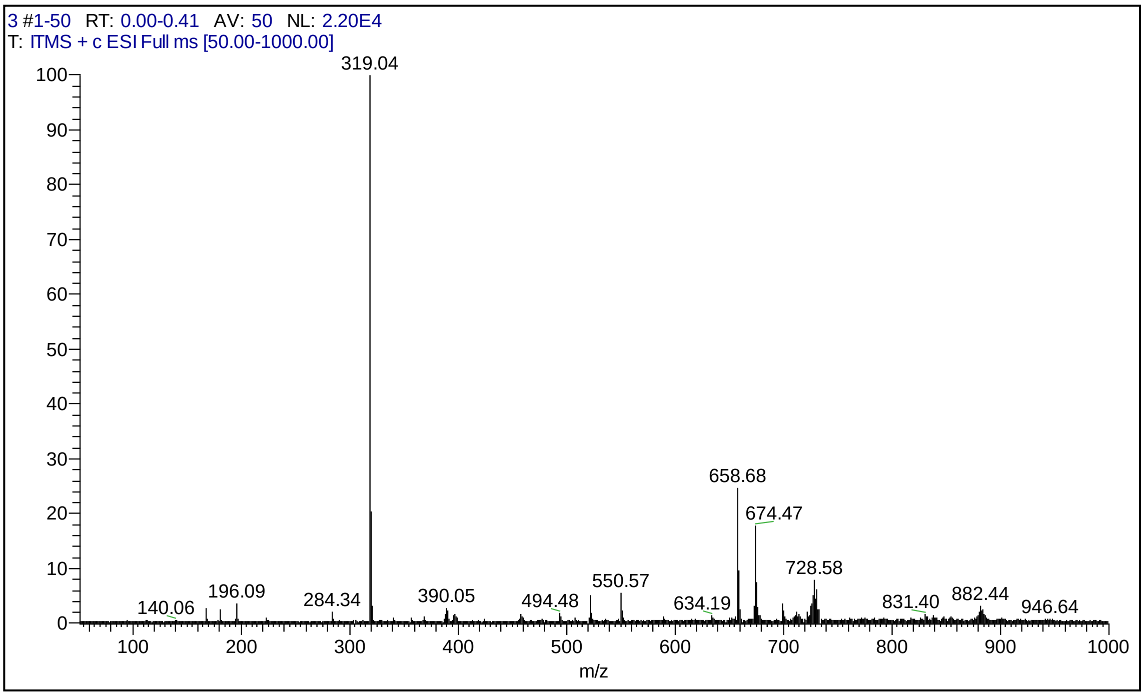

Figure S8. ESI-MS spectrum of **L²** in MeOH:H_2O (1:1).

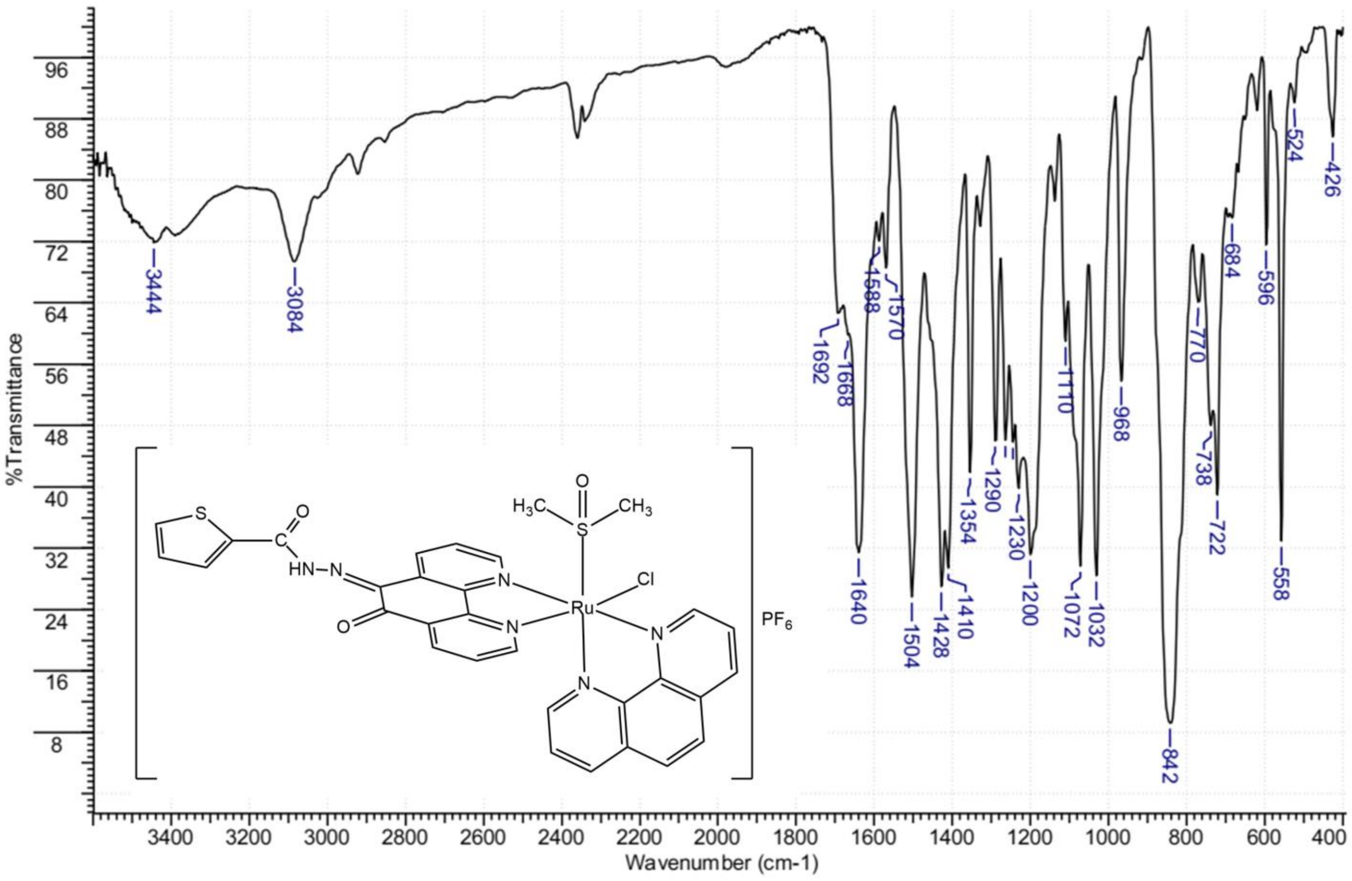

Figure S9. Infrared spectrum of complex **1**.

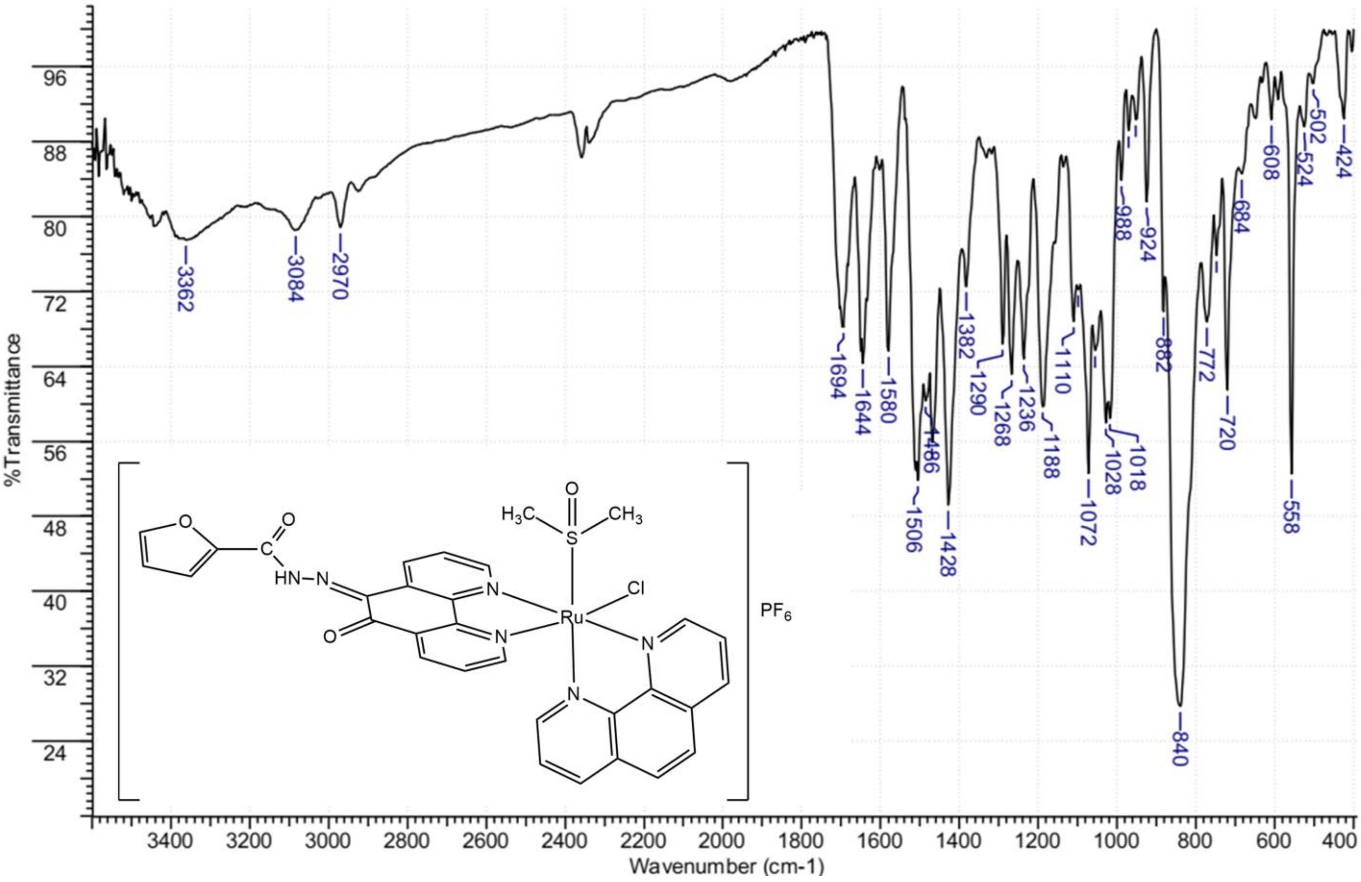

Figure S10. Infrared spectrum of complex **2**.

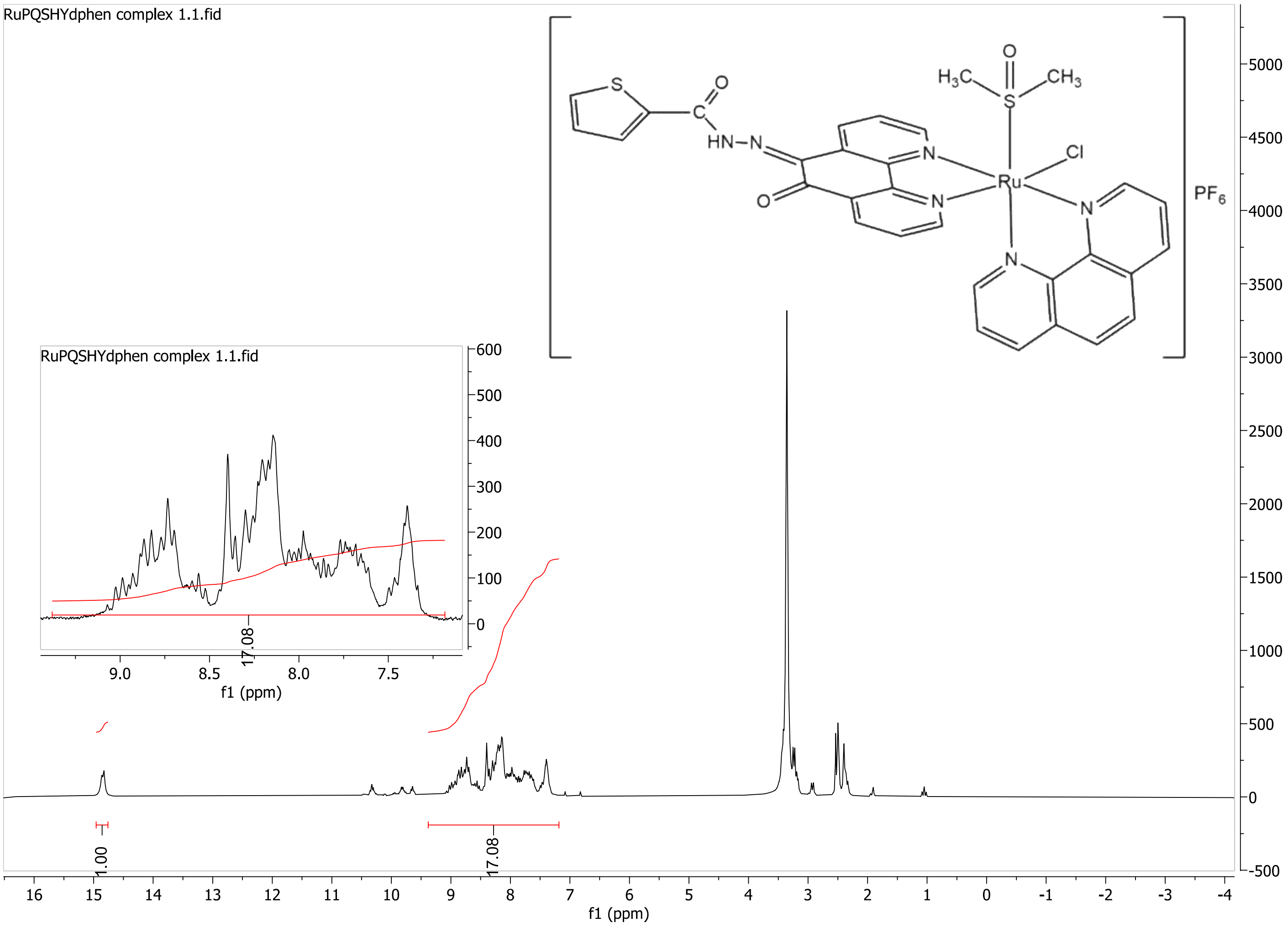

Figure S11. 1H NMR spectrum of complex **1** (200 MHz) in dmso-d6.

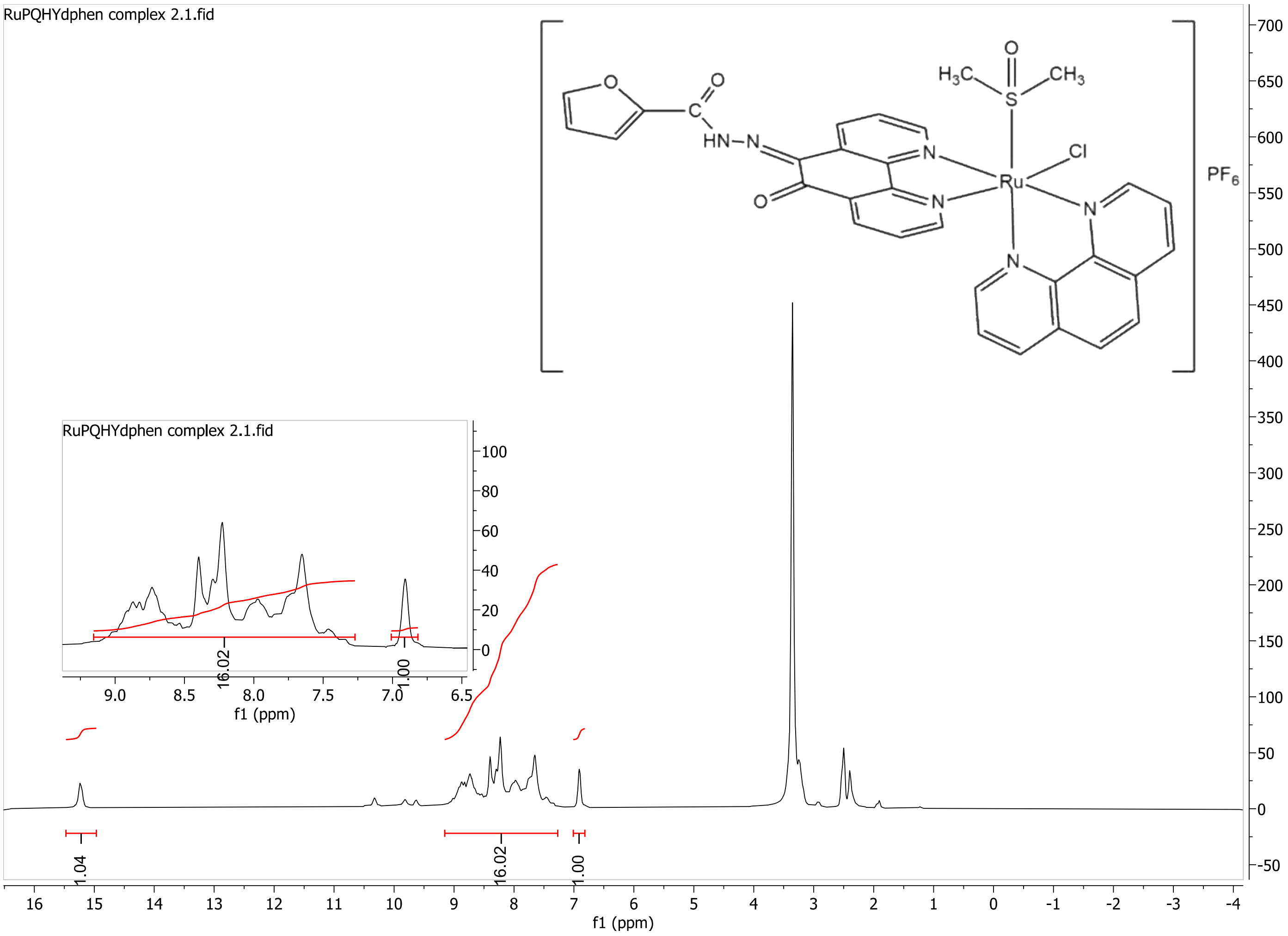

Figure S12. ^1H NMR spectrum of complex **2** in dmso-d6.

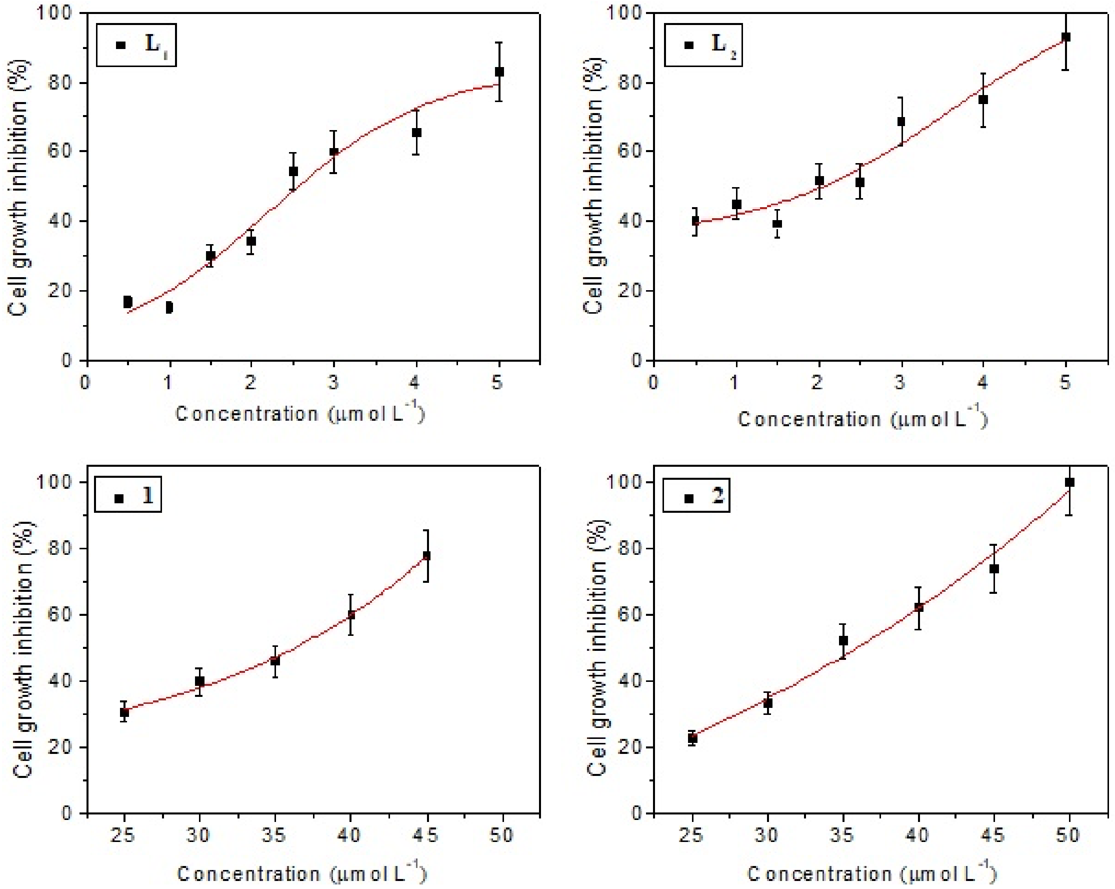

Figure S13. Dose-response curves for L[1], L[2], complex **1** and complex **2**. K562 cells were incubated with different concentrations of tested compound for 72h. The values are the average of three independent experiments.

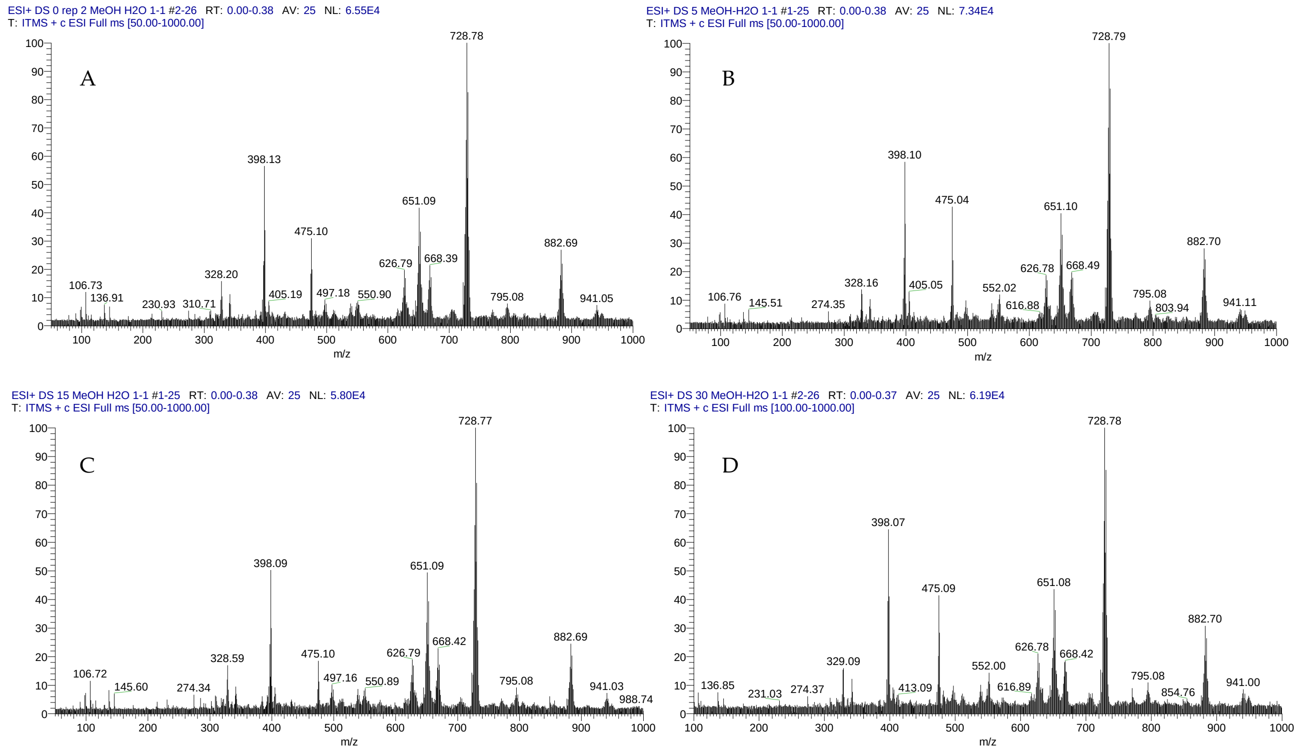

Figure S14. Mass spectra (ESI-MS) of complex **1** before (A) and after UV-A light irradiation (B, C, D) for 5, 15, and 30 min, respectively.

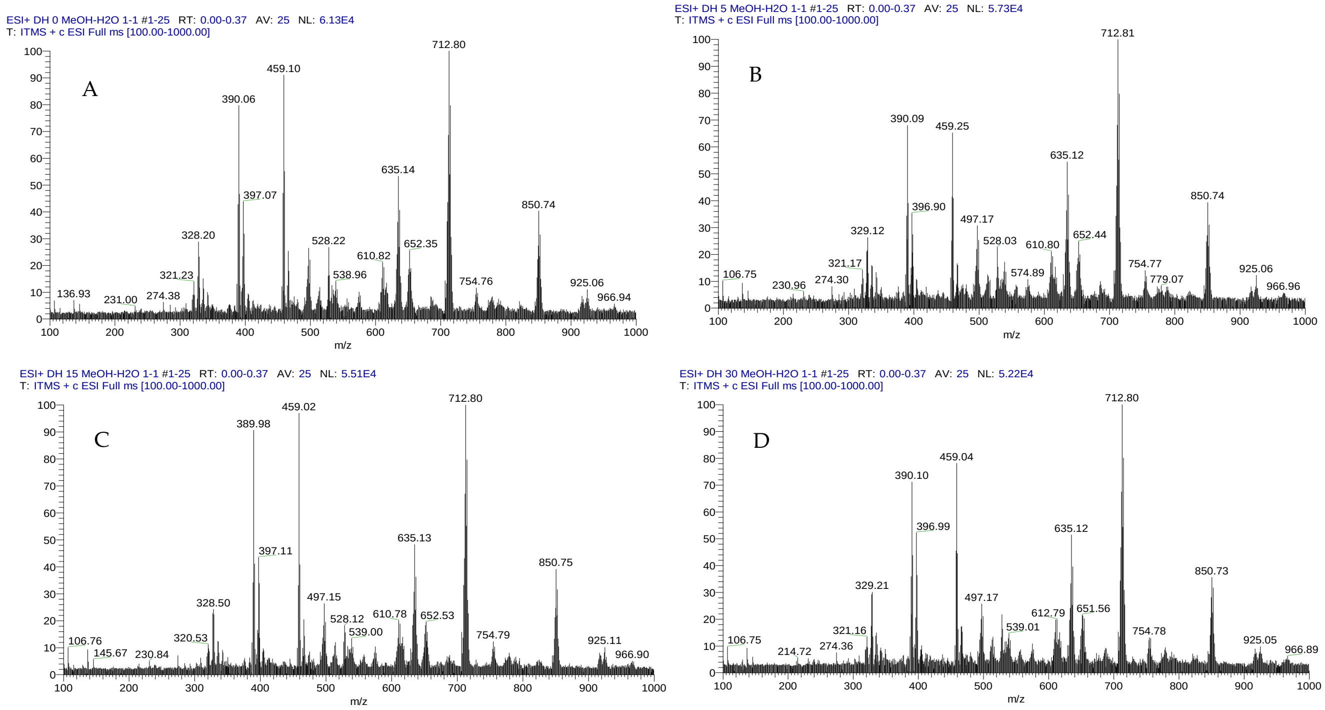

Figure S15. Mass spectra (ESI-MS) of complex **2** before (A) and after UV-A light irradiation (B, C, D) for 5, 15, and 30 min, respectively.

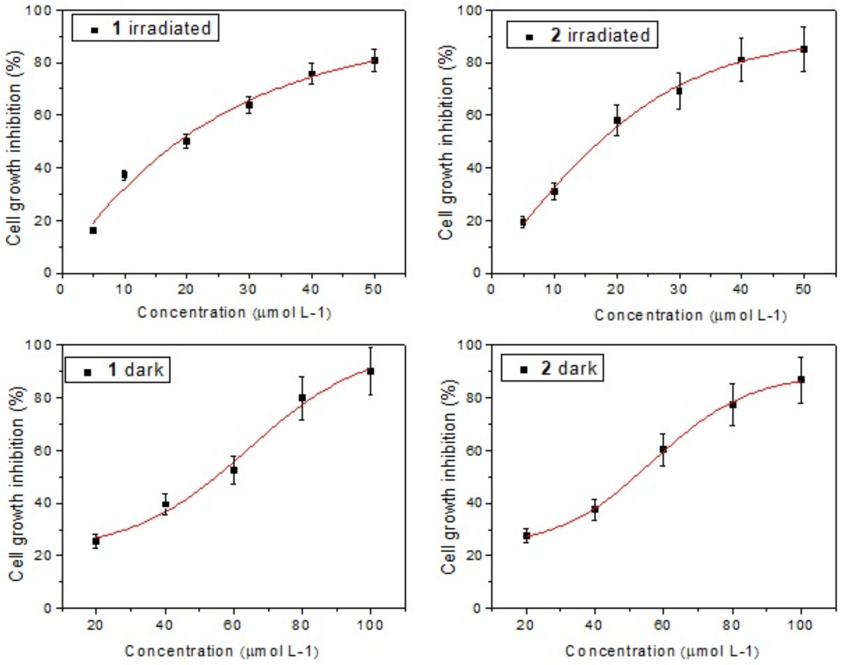

Figure S16. Photocytotoxic assays for complex **1** and complex **2**. K562 cells were incubated with different concentrations of tested compound for 4 h in the presence of different complex concentrations, in the dark and after 5 min of UV-A light exposure. The values are the average of three independent experiments.

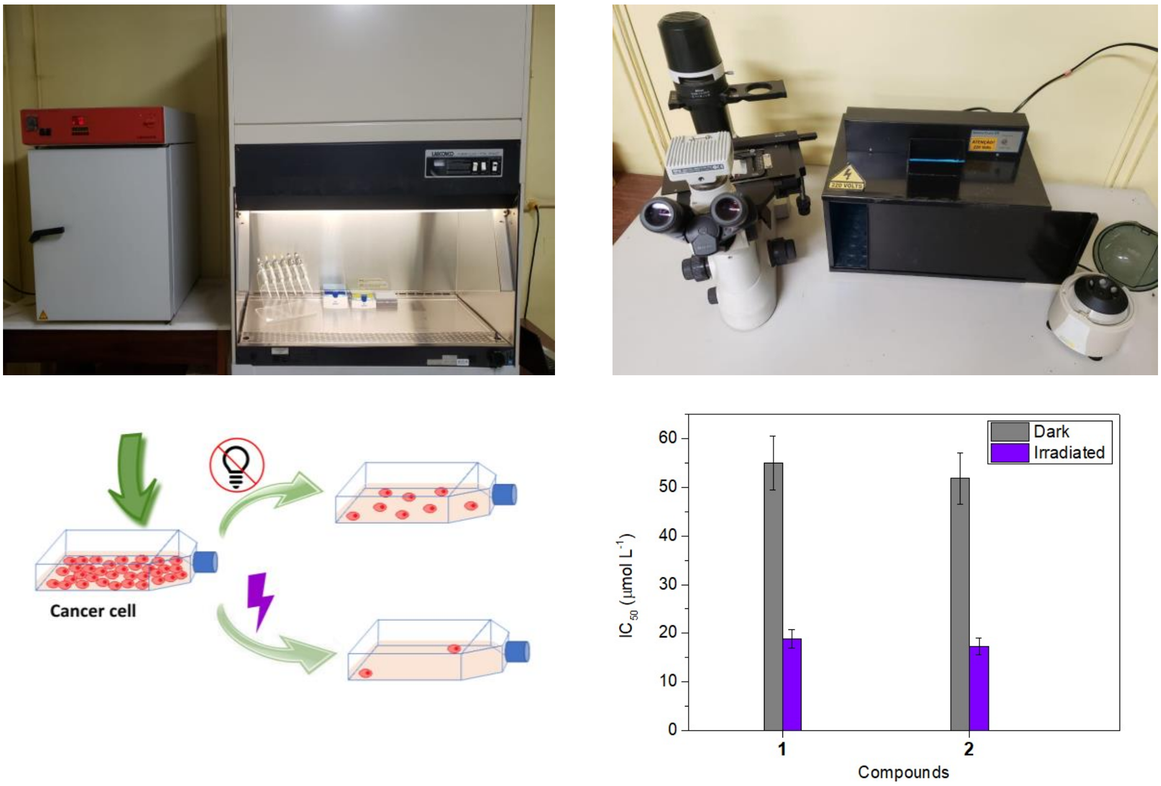

Sketch for the photocytotoxicity assay.

1 × 10^5 cells mL^{-1} were cultured in the dark for 4h in the absence and the presence of a range of concentrations of tested compounds. Subsequently, cells were washed three times with ice-cold phosphate-buffered saline (PBS) to eliminate the culture medium. After replacement of the culture medium with PBS, the cells were photoirradiated with UV-A light (365 nm) for 5 min in an ultraviolet fluorescence cabinet. After irradiation, PBS was replaced with RPMI 1640 medium supplemented with 10% fetal calf serum, and incubation was continued for a further 72h in the dark. For cytotoxic and photocytotoxic assays, the sensitivity to compound was evaluated by the concentration that inhibits cell growth by 50%, IC_{50}.
